# A Review—Additive Manufacturing of Intermetallic Alloys Based on Orthorhombic Titanium Aluminide Ti_2_AlNb

**DOI:** 10.3390/ma16030991

**Published:** 2023-01-20

**Authors:** Anatoliy G. Illarionov, Stepan I. Stepanov, Inna A. Naschetnikova, Artemiy A. Popov, Prasanth Soundappan, K. H. Thulasi Raman, Satyam Suwas

**Affiliations:** 1 Heat Treatment & Physics of Metals Department, Ural Federal University Named after the First President of Russia B.N. Yeltsin, 19 Mira St., 620002 Ekaterinburg, Russia; 2 M. N. Mikheev Institute of Metal Physics, 18 S. Kovalevskaya St., 620108 Ekaterinburg, Russia; 3 Department of Materials Engineering, Indian Institute of Science, Bangalore 560012, India; 4 Society for Innovation, Indian Institute of Science, Bangalore 560012, India

**Keywords:** Ti_2_AlNb-based alloy, metal additive manufacturing, microstructure, mechanical properties, phase transformation

## Abstract

Titanium alloys based on orthorhombic titanium aluminide Ti_2_AlNb are promising refractory materials for aircraft engine parts in the operating temperature range from 600–700 °C. Parts made of Ti_2_AlNb-based alloys by traditional technologies, such as casting and metal forming, have not yet found wide application due to the sensitivity of processability and mechanical properties in chemical composition and microstructure compared with commercial solid-solution-based titanium alloys. In the last three decades, metal additive manufacturing (MAM) has attracted the attention of scientists and engineers for the production of intermetallic alloys based on Ti_2_AlNb. This review summarizes the recent achievements in the production of O-phase-based Ti alloys using MAM, including the analysis of the feedstock materials, technological processes, machines, microstructure, phase composition and mechanical properties. Powder bed fusion (PBF) and direct energy deposition (DED) are the most widely employed MAM processes to produce O-phase alloys. MAM provides fully dense, fine-grained material with a superior combination of mechanical properties at room temperature. Further research on MAM for the production of critical parts made of Ti_2_AlNb-based alloys can be focused on a detailed study of the influence of post-processing and chemical composition on the formation of the structure and mechanical properties, including cyclic loading, fracture toughness, and creep resistance.

## 1. Introduction

Titanium has two allotropic forms in its solid state and belongs to the class of light metals. The low-temperature α-Ti (HCP) modification transforms into a high-temperature β-Ti (BCC) modification at 882.5 °C [1]. Titanium is alloyed with α-stabilizing elements (Al, O, N and C), which increase the β-transus temperature (β_tr_), β-stabilizing elements (Mo, V, Nb and Ta) and eutectoid-forming elements (Fe, Cr, Mn, Ni and Si), which reduce β_tr_ and neutral elements (Zr, Sn and Hf) that have little effect on β_tr_. Depending on the content of alloying elements and the phase composition formed during processing, titanium alloys are classified into α-, near-α-, (α + β), near-β and β-alloys, as well as intermetallic alloys based on Ti_3_Al (α_2_), Ti_2_AlNb (O) and TiAl (γ) [1,2].

**Figure 1:**
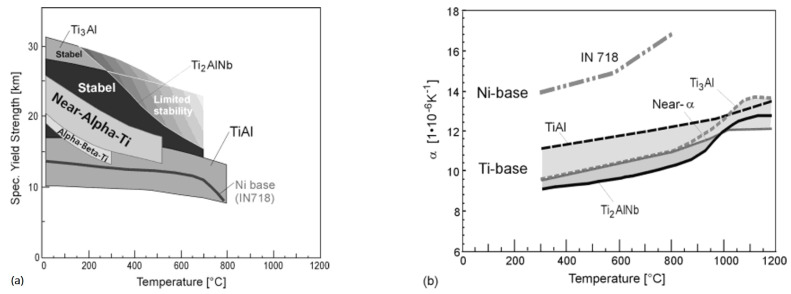
Temperature dependences of specific yield strength (**a**) and coefficient of thermal expansion (**b**) for orthorhombic titanium aluminide (Ti_2_AlNb and Ti-22Al-25Nb alloy) compared with near-α-titanium alloys (Timetal 834/1100), Ti_3_Al-based alloy (Ti-25Al-10Nb-3V-1Mo), TiAl-based alloy (Ti-46.5Al-3.0Nb-2.1Cr-0.2W) and nickel-based alloy (IN718). Reproduced with permission from Elsevier [4].

Intermetallic titanium alloys based on Ti_2_AlNb have attracted the attention of scientists and engineers in the past three decades since D. Banerjee and co-authors discovered the presence of a new phase (O-phase) with an orthorhombic crystal lattice and the thermodynamic conditions for its formation in the Ti-25Al-12.5Nb alloy (at. %) [3]. Intermetallic alloys based on orthorhombic titanium aluminide have higher specific strength (σ_0.2_/g_ρ_) at elevated temperatures compared with refractory near-α- and (α + β)-alloys (Figure 1a). Therefore, such alloys are considered promising for refractory materials in the temperature range from 600–700 °C for aircraft engines instead of heavier nickel-based alloys [4].

In addition, intermetallic alloys based on orthorhombic titanium aluminide compete with refractory alloys based on titanium aluminides Ti_3_Al (α_2_-phase) and TiAl (γ-phase) due to a lower coefficient of thermal expansion (Figure 1b), higher ductility and workability and fracture toughness at room temperature [4,5]. Additionally, alloys based on titanium aluminide Ti_3_Al are characterized by strong embrittlement due to active oxidation at temperatures above 550 °C [4]. Nowadays, their introduction in the industries has slowed down. Scientific and practical interest has been shifted towards intermetallic alloys based on orthorhombic titanium aluminide [4].

Despite the several advantages of intermetallic alloys based on orthorhombic titanium aluminide as refractory materials, the production of semi-finished products by traditional technologies (casting and metal forming) has not yet found the necessary development. Shape casting of intermetallic alloys based on orthorhombic titanium aluminide is not currently used due to several unresolved problems associated with the following aspects: (1) segregation of the main alloying elements (Al and Nb) that leads to chemical and phase inhomogeneity over the cross-section of the ingot; (2) shrinkage porosity; (3) unfavorable crystallization texture; (4) and the formation of a coarse-grained structure [6]. The main reason for the noted issues is a significant difference in the physical properties of the base material and alloying elements, especially the density and melting temperatures of Al (660 °C) and Nb (2468 °C) [7].

The above-mentioned problems with casting lead to manufacturing difficulties in obtaining high-quality deformable, semi-finished products and welded joints [6]. In particular, manufacturability (plasticity, machinability and weldability) and mechanical (strength, ductility, fracture toughness, etc.) properties of intermetallic alloys based on orthorhombic titanium aluminide are more sensitive to composition and structure compared to commercial titanium alloys based on solid solutions [4,6]. As a result, there is a lack of data on the manufacturing conditions of forged and stamped parts of O-phase alloys Ti-24Al-15Nb [8] and Ti-22Al-25Nb used in actual engine applications.

Powder metallurgy is an alternative way of producing intermetallic alloys based on orthorhombic titanium aluminide [6,9]. It allows the manufacturing of products with complex shapes and sufficient properties, especially in combination with hot isostatic pressing (HIP) and heat treatment (HT). However, there are aspects associated with the residual porosity of the semi-finished product. As a result, weldability and lifetime under cycling loading may decrease since pores can initiate fatigue cracks.

Nowadays, additive manufacturing (AM) gains more interest in obtaining intermetallic alloys based on orthorhombic titanium aluminide [10,11,12,13,14]. Innovative results on the AM of titanium alloys have already been achieved for titanium alloys with special functional properties, e.g., biomedical applications [15] and shape memory alloys [16,17]. The first results of additive manufacturing of refractory TiAl-based alloys are discussed in reviews [18,19]. However, there are still no comprehensive reviews on the AM of O-phase-based Ti alloys, including an analysis of the materials used, process and machines/equipment characteristics, structure formed during processing, phase composition and properties. This review aims to fill this gap.

## 2. General Characteristics of Alloys Based on Ti_2_AlNb: Phases, Phase Diagrams, Alloying Elements, Microstructure and Properties

**Figure 2:**
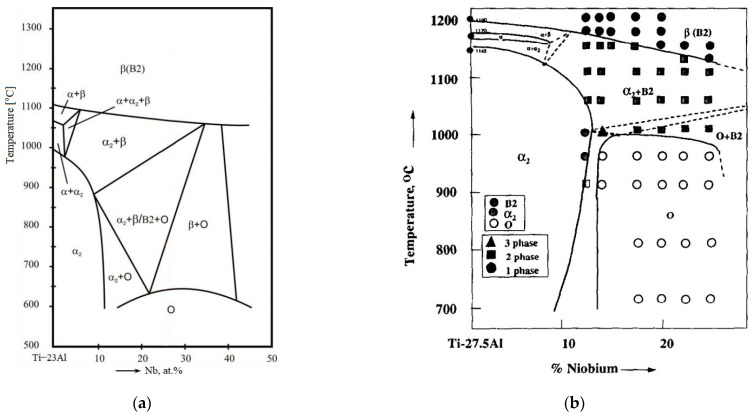
Isopleth sections of the (**a**) ternary Ti-23%Al-xNb diagram [22] and (**b**) Ti-27.5% Al-xNb. Diagram reproduced with permission from Elsevier [24].

Titanium alloys containing the O-phase developed based on the Ti-Al-Nb system usually contain 18–30% of Al and 12.5–30% of Nb [4,20]. The equilibrium phase composition of O-phase alloys is based on the isopleth sections of the Ti-Al-Nb phase diagram, which corresponds to constant Al content (22, 23, 25, 27.5 at%) and varied Nb content [21,22,23,24]. Diagrams for alloys with 23 and 27.5% Al are shown in Figure 2.

According to the diagram, depending on the content of Al and Nb, different phases get stabilized at different temperature regimes. Phases available in different regimes include:

− Ordered (B2) and disordered (β) solid solution based on the BBC lattice;
− α_2_-phase based on intermetallic Ti_3_Al with ordered HPC lattice;
− Ordered O-phase-based on Ti_2_AlNb with orthorhombic lattice, which is divided into two types in [24]: The 1st type (high-temperature O-phase)—Al ordered and Ti and Nb disordered—O_1_-phase, the 2nd type (low-temperature)—Ti, Al and Nb ordered—O_2_-phase. Most diagrams have no such division and use the generalized name O-phase.

**Figure 3:**
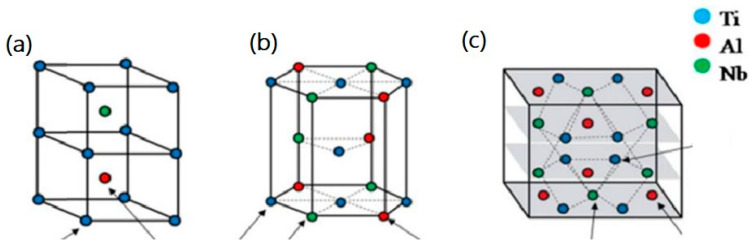
Crystal structures of (**a**) B2, (**b**) α_2_ and (**c**) O phase with Wyckoff positions and occupancies. Reproduced with permission from Elsevier [25].

The crystal lattices of the ordered B2, α_2_ and O_2_ phases are shown in Figure 3.

By analogy with alloys based on titanium solid solutions, alloying elements in alloys based on orthorhombic titanium aluminide can be divided into α-stabilizing elements (Al), β-stabilizing elements (Nb, Mo, V, Ta, Fe, Si and H) and neutral elements (Zr, Hf, B and Y). To estimate the composition of the multicomponent O-phase alloy in the Ti-Al-Nb phase diagrams, the so-called structural equivalents are utilized by analogy with titanium alloys based on solid solutions. The structural Al equivalent ([Al]_eq_) allows us to estimate the influence of the alloying elements and impurities on the stability of the α(α_2_) phase relative to the corresponding influence of Al. This equivalent of α-stabilizing elements and neutral elements, introduced by Rosenberg [26], is described by the following ratio:

[Al]_eq_ = %Al + %Sn/3 + %Zr/6 + 10%O (mass.%). (1)

Since these alloys can be doped with other β-stabilizers, the concept of the Nb equivalent ([Nb]_eq_) can be employed. [Nb]_eq_ can be estimated by two methods. The first method [4] proceeds from the fact that the stabilizing elements in the O-phase occupy the Nb lattice position, and according to this method, 1% of any β-stabilizing element is equivalent to 1% of Nb. The second method [22] considers the effect of β-stabilizers on the stability of the high-temperature β-solid solution in O-phase alloys by analogy with their effect on the stability of the matrix β-phase in solid solution-based titanium alloys during quenching. It takes into account the critical concentrations for binary alloying systems (by analogy with [Mo]_eq_ [2]), above which only the β-solid solution is present and the martensitic transformation in the alloy is suppressed. In [22], the following critical concentrations (wt.%) were considered: 36 Nb; 11 Mo; 15 V; 45 Ta; 22 W; 4.5 Fe; 6.5 Cr; 6.5 Mn; 9.5 Co; and 8.5 Ni. Based on these values for O-alloys, the structural [Nb]_eq_, which evaluates the effect of each β-stabilizing element in the alloy on the stability of the β-solid solution relative to Nb, can be calculated by the formula [22]:

[Nb]_eq_ = %Nb + %Mo/0.31 + %V/0.42 + %Ta/1.25 + %W/0.61 + %Fe/0.13 + %Cr/0.18 + %Mn/0.18 + %Co/0.26 + %Ni/0.24 (2)

The mechanisms and stages of phase transformations (β(B_2_)-O, α_2_-O, β(B2)-α_2_ and β(B_2_) + α_2_-O in O-phase alloys at constant temperature (during aging or holding after quenching at annealing temperatures) are addressed in quite a few studies and their detailed analysis is given in reviews [25,27,28,29]; therefore, we shall not dwell on this topic in our review.

**Figure 4:**
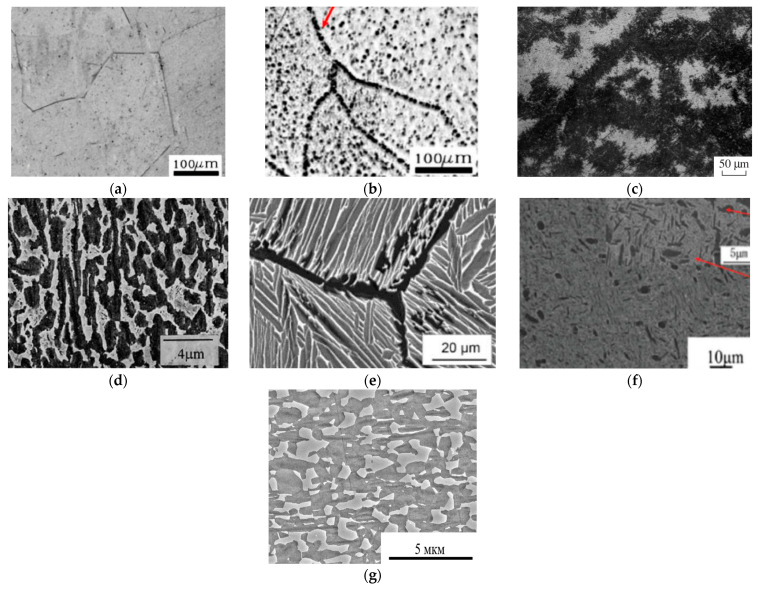
Typical microstructures of O-phase alloys: (**a**) Single-phase β(B2) with granular β(B2)-structure obtained by holding above β_tr_ at 1100 °C for 30 min and water quenching of Ti-22Al-25Nb O-phase alloy [30]; (**b**) two-phase β(B2) + α_2_ state with α_2-_precipitates at the boundaries and within β(B2) grains after quenching of Ti-22Al-25Nb alloy from (β(B2) + α_2_)-region at 1020 °C from [30]; (**c**) two-phase β(B2) + O state with lamellar structure of conglomerates of thin O-phase plates on the boundaries and within β(B2) grains after quenching of cast alloy VTI-4 from (β(B2) + O)-region at 700 °C [31]; (**d**) two-phase β(B2) + O state with the structure of light deformed O-phase platelets in the dark β(B2)-matrix, obtained by hot deformation of Ti-22Al-27Nb alloy at temperatures below β_tr_ [32]; (**e**) three phase β(B2) + α_2_ + O state with lamellar structure with thick grain boundary α_2-_plates and coarse secondary O-phase plates on the boundaries and within β(B2) grains after slow cooling from heating temperatures above β_tr_ of Ti-22Al-25Nb [4]; (**f**) three-phase bimodal structure of equiaxed dark primary α_2_-precipitates (indicated by upper arrow) and packets of thin O-phase lamellae (indicated by lower arrow) in β(B2)-matrix obtained in the Ti-22Al-25Nb by forging at 1020 °C followed by solid solution treatment at 960 °C and aging at 780 °C [30]; (**g**) two-phase β(B2) + O state with equiaxed O-phase structure in β(B2)-matrix obtained in the Ti-23Al-27Nb by quenching from 900 °C after hot rolling in (β(B2) + O)-region. Reproduced with permissions from John Wiley and Sons [4], Elsevier [27,29] and Springer [31].

The type of structure formed in O-phase alloys during deformation and heat treatment is determined by the temperature ranges and cooling rates used during processing and can result in single-phase β(B_2_), two-phase (β(B2) + O) or three-phase (β(B2) + α_2_ + O) microstructures with different morphologies of O (α_2_)-precipitates, namely lamellar, equiaxed and globular-lamellar (duplex) structures and also different levels of grain refinement (Figure 4). Obtaining different structural phase states is possible by varying the temperature rate parameters of heat treatment and deformation processing.

According to [3,21,33,34,35,36,37,38,39,40,41,42,43], a favorable effect on ductility and impact toughness of O-phase alloys results from an increase in the volume fraction and refinement of β(B2)-grain size, the absence of grain boundary α_2_-precipitates and thickening of O-phase plates. An increase of secondary O-phase volume fraction along with a reduction of α_2_-phase and the formation of refined O-phase platelets enhance strength characteristics.

The influence of chemical composition, deformation processing of O-phase alloys with various types of structure and range of mechanical properties is discussed in detail in [44]. It was shown that in O-phase alloys, such as Ti-22Al-25Nb, after isothermal forging and subsequent strengthening heat treatment (quenching and aging), the following range of properties can be achieved: UTS = 1040–1172 MPa, YS = 875–1065 MPa; EL = 6.5–14.8; RA = 11–15.3%.

## 3. Feedstock Materials

To produce parts from alloys based on Ti_2_AlNb by AM, either wire or powder are utilized. Due to the limited ductility of the Ti_2_AlNb-based alloys [4], the simultaneous feeding of two wires made of pure Al and a binary Ti-Nb alloy is employed in WAAM. The composition of the Ti-Nb wire is set so that the orthorhombic titanium aluminide of a given composition is fused. The characteristics of the wire used are given in Table 1.

The following types of powders can be used for the AM of Ti_2_AlNb-based alloys:

(1) Elemental powders of pure Ti, Al, Nb and other metals [12,45,46,47,48,49];
(2) Elemental powders of pure Ti and pre-alloyed Al-Nb [50];
(3) Pre-alloyed powders of a given composition [10,11,12,13,51,52,53,54,55,56,57,58,59,60,61,62].

**Figure 5:**
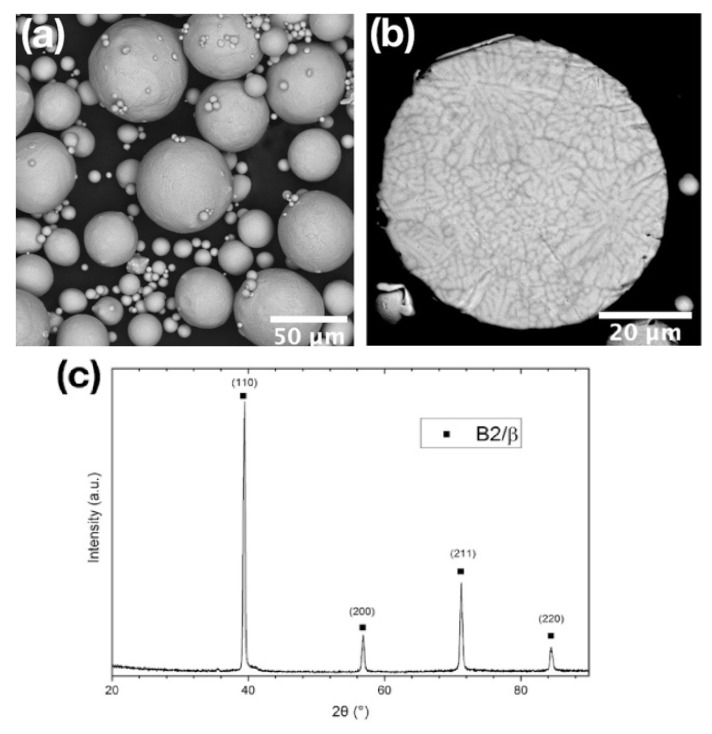
SEM images of the Ti_2_AlNb-based powder showing (**a**) particles’ surface morphology; (**b**) their cross-section; (**c**) XRD pattern of the powder. Reproduced with permission from Elsevier [72].

The characteristics of the feedstock powders are summarized in Table 2, and their SEM images are presented in Figure 5. A literature analysis showed that for additive manufacturing, the spherical Ti_2_AlNb-based powders are produced by the electrode induction gas atomization (EIGA) method with particles size in the range of 15–60 μm, by the plasma atomization (PA) method and by the plasma rotating electrode atomization (PREP) method with particles size in the range of 40–200 µm. In [63], the possibility of producing orthorhombic titanium aluminide powders by the hydrogenation–dehydrogenation method was reported. However, these powders have not yet been used for additive manufacturing due to their non-spherical shape. In addition, from 2008 to 2018, only Ti-22Al-25Nb powders were produced, and since 2018, more complex compositions that include Mo [20,64,65,66] and, in some cases, V, Zr, Si and Hf have been reported [12,55,56]. Note that the best combination of mechanical properties of AM orthorhombic titanium aluminides was obtained using pre-alloyed powders, rather than elemental powders, apparently due to the additional operations needed (mixing, spheroidization, mechanical alloying, etc.) to be performed prior to the fusion of pure metals, powders and alloys. These operations may lead to contamination with impurities, primarily oxygen, which contribute to the embrittlement of the AM part.

**Table 1:** The nominal chemical composition of Ti-Nb wire and theoretical composition of the Ti_2_AlNb-based alloy fused using AM process, diameter and feed rate of the wire.

Wire Materials	Chemical Composition of Components [wt.%]	Wire Diameter [mm]	Wire Feed Rate, [m/min]	Alloy Theoretical Composition [at.%]	Ref.
Al Ti-Nb	Al > 99.7	1.6	0.3	Ti-24.8Al-22.3Nb	[67]
	Ti-45.8Nb-0.08Fe-0.06C-0.023N-0.12O-0.011H	1.2	1.7		
Al Ti-Nb	Al > 99.999	1.6	0.57	Ti-24.8Al-23.5Nb	[14]
			0.6	Ti-25.7Al-23.2Nb	
	Ti-46.94Nb-0.01Fe-0.012C-0.008N-0.048O-0.0008H	2.0	0.63	Ti-26.7Al-22.9Nb	
			0.51	-	
			1.18	-	

In addition, the mechanically alloyed and plasma spheroidized (MAPS) method for the production of powders may contribute to an increase of Fe (the eutectoid-forming β-phase stabilizing element) due to the wearing of the grinding balls used for mechanical alloying [12].

**Table 2:** Characteristics of the feedstock powders used for AM of Ti_2_AlNb based alloys.

Powder Materials	Chemical Composition of Components	Powder Size [µm]	Production Methods		Alloy Chemical Composition [at. %]		Refs.
			Powder	Part	Theoretical	Experimental	
cp-Ti Grade 2	Ti > 99.6 wt.%	d_10_ = 23.8 d_50_ = 44.6 d_90_ = 73.1	-	SLM (BJ)	Ti-22Al-25Nb	-	[45,46,48,49,68]
Pure Al	Al > 99.9 wt.%	d_10_ = 8.5 d_50_ = 21.2 d_90_ = 41.1					
Pure Nb	Nb > 99.7 wt.%	d_10_ = 15.1 d_50_ = 32.9 d_90_ = 65.1 *					
Pure Ti + Al-Nb	99.99 Ti wt.% weight ratio Al/Nb = 22/75	38 44	-	LSF	Ti-20Al-27Nb Ti-22Al-27Nb	-	[50]
Mixture of pure Ti, Al, Nb, Mo, Zr, Si, Hf, Ta	99.9%	d_10_ = 24.0 d_50_ = 63.3 d_90_ = 98.5	MAPS	L-PBF	Ti-22Al-25Nb-0.3Mo-0.2Hf-0.4Ta-1Zr-0.3Si	Ti-16Al-22Nb-0.1Mo-0.3Hf-0.3Ta-1.5Zr-0.8Si-0.9Fe	[12]
Ti-Al-Nb	Ti–9.54Al–42.24Nb wt.%/	150–212	-	LSF	Ti_2_AlNb	-	[57,58]
Ti-Al-Nb	Ti–9.68Al-41.37Nb-0.05V-0.0079O- 0.053N-0.0024H wt.%	38–160	PREP	LMD	Ti-22Al-25Nb	-	[51,59,60,61,62]
Ti-Al-Nb	-	38–160	PREP	LAM	Ti-22Al-25Nb	-	[10,52,53]
Ti-Al-Nb	Ti-19.59Al-24.32Nb- < 0.4V-0.18O-0.02N-0.13H at%	60–185	PA	LDM	Ti-19.59Al-24.32Nb- < 0.4V-0.18O-0.02N-0.13H	-	[69]
Ti-Al-Nb	Ti-22.78Al-24.83Nb-0.1104O-0.0282N at%	d_10_ = 15.9 d_50_ = 32.5 d_90_ = 58.2	EIGA	SLM	Ti-22.78Al-24.83Nb	Ti-18.58Al-25.59Nb	[11,13,70]
Ti-Al-Nb	Ti-11.24Al-43.8Nb-0.06Fe-0.38O-0.15C-0.003N wt.%	53–150	EIGA	SEBM	Ti-22Al-25Nb	Ti-10.04Al-44.08Nb (wt.%)	[71]
Ti-Al-Nb	Ti-22.13Al-24.95Nb- 0.03O-0.02N at%	40–160 dav. = 90.85	PA	LD	Ti-22.13Al-24.95Nb- 0.03O-0.02N	Ti-21.18Al-25.37Nb-0.12O-0.1N	[54]
			PA	PF-LD	Ti-22.13Al-24.95Nb-0.03O-0.02N	Ti-21.21Al-25.35Nb-0.11O-0.08N	
Ti-Al-Nb-Mo	Ti–21.68Al–25.02Nb-0.59Mo at%	15–53	-	SLM	Ti-22Al-24Nb-0.5Mo	Ti-21.24 Al-24.78Nb-0.57Mo	[64]
						Ti-20.63Al–25.04 Nb-0.57Mo	
						Ti-20.09Al-25.87Nb-0.61Mo	
Ti-Al-Nb-Mo	Powder: Ti-9.44Al-39.31Nb-3.45Mo wt.%	40–105	PREP	LAW	Ti-22Al-25Nb	Weld: Ti-10.46Al-39.76Nb-1.61Mo (wt.%)	[20,65,66]
	Base metal: Ti-9.3Al-38.16Nb-0.9Mo wt.%						
Ti-Al-Nb-Zr-V-Mo-Si	Ti-24Al-25Nb-1Zr-1.4V-0.6Mo-0.3Si at%	d_10_ = 14.6 d_50_ = 29.3 d_90_ = 52.3	EIGA	L-PBF	Ti-24Al-25Nb-1Zr-1.4V-0.6Mo-0.3Si	-	[12,55,56]

* non-spherical powder.

## 4. Classification of the MAM Processes Used for Ti_2_AlNb Based Alloys

Due to the struggle for primacy for the opportunity to benefit from intellectual property rights, the development of additive manufacturing in the last three decades has become so rapid that it led to the exponential growth of the research, software products, AM machines, materials and post-processing technologies [73]. As a result, these outcomes are hardly amenable to systematization and classification. Moreover, the complexity of technologies tends increase in order to meet the main challenges of additive manufacturing: porosity due to powder materials use [74], residual stresses due to significant heating and cooling rates [75], the requirements for surface roughness [76] and tailoring the operational properties for different applications (namely bearing [77], magnetic [78], biomedical [79,80], refractory [19], etc.). At present, there are about 30 different metal additive manufacturing (MAM) processes. Thus, to summarize and classify the MAM processes three main categories are employed: working principle or MAM category, feedstock material and energy source (Table 3).

According to EN ISO/ASTM 52900 (2021), standard additive manufacturing processes are classified into seven categories, only four of which are utilized to build metal parts: binder jetting (BJ), powder bed fusion (PBF), sheet lamination (SL) and direct energy deposition (DED). The other three categories (vat photopolymerization (VP), material jetting (MJ) and material extrusion (ME)) are mainly used for ceramics, composites, polymers and resins or as indirect additive manufacturing to provide machine–tool attachment for traditional manufacturing processes [81]. The feedstock materials are used in MAM are powder, wire and sheets (foils). The source of feedstock material processing includes laser, electron beam, ultrasonic welding head, wire arc and liquid binding agent combined with furnace heating. Schematic representation of the working principle of each of them have been repeatedly presented earlier [81], and in this work, the emphasis is on the basic technical features of the MAM processes (Table 4).

Further detailed exploration of AM processes can be made by the analysis of the process parameters; more than 130 parameters of the selective laser melting (SLM) process alone are distinguished in [82]. The key technological parameters, including laser power, hatching distance, volume energy density, layer thickness and scanning speed, determine the specific energy that provides the synthesis of feedstock material and is summarized in Table 3. In addition, further AM enhancement is possible due to employment of hybrid additive manufacturing (HAM) [81], which combines AM with each other or AM with traditional processing technologies: machining, deformation, magnetic and ultrasonic treatments, etc.

### 4.1. Powder Bed Fusion (PBF)

In powder bed fusion additive manufacturing, powder is applied with a recoater to the platform layer by layer. A thermal energy source, such as a laser or electron beam, fuses the powder before the next layer is applied. Laser powder bed fusion (L-PBF) manufacturing occurs in controlled environment atmospheres of argon or nitrogen with a deposition rate in the range from 2–20 sm^3^/h. The surface roughness of L-PBF parts lies in the range from 10–20 μm [81]. Optionally, preheating the base platform up to 1000 °C can be applied [64].

Electron beam powder bed fusion (EB-PBF) takes place under controlled vacuum conditions. The electron beam is focused and deflected by means of an electromagnetic lenses, instead of mirrors as in L-PBF. The deposition rates typical of EB-PBF correspond to the range from 55–80 sm^3^/h and provide surface roughness up to the values in the range from 15–30 μm [81]. Pre-heating each layer by scattered electron beam to temperatures about 0.6 of the melting temperature is required to avoid so-called ‘smoking’ [83].

There are some disadvantages to the powder bed fusion method related to the efficiency of the process both for the transformation power from electricity to laser and for the production output rate. The main advantages are the good resolution, reduced material wastage and efficient recycling of the un-melted powder.

### 4.2. Direct Energy Deposition (DED)

In direct energy deposition or direct metal deposition (DMD), a four- or five-axis arm moves around, depositing melted material around a fixed object. An electron beam, wire arc or laser melts the powder or wire feedstock adding the material to the substrate. Simultaneous deposition and melting during the construction of the parts should be provided. In the case of metal, a powder is coaxially fed through the laser head and provides a much better finish than wire. In contrast, an independent feeding system in the case of wire is employed. The DED deposition rate lies in the range from 20–160 sm^3^/h, and surface roughness is typically above 30 μm [81]. The desired effect with the wire can be achieved through post-processing. The problem of significant thermal stresses arises due to the high deposition rates and large melt pools.

In laser direct energy deposition (L-DED), the shielding gas’ flow head protects the melt pool from oxidation and carries the powder to the melt pool. The electron beam direct energy deposition (EB-DED) works exclusively with wire as a feedstock due to poor powder flow in a vacuum.

WAAM (wire arc additive manufacturing) combines principles similar to arc welding processes and the digital control of electric arc displacement. WAAM-based processes are more efficient than L-DED due to the larger energy requirements. Machining to obtain the final parts produced by EB-DED and WAAM is assumed; therefore, the surface roughness is irrelevant.

GMA-DED is the simplest, cheapest and most widely spread process to implement due to its direct wire-feeding, which is coaxial with the nozzle of the welding torch. In gas tungsten arc direct energy deposition (GTA-DED) [84] and plasma arc direct energy deposition (PA-DED) [85], non-consumable electrodes generally made of tungsten are used. The feedstock wire is supplied through an additional wire-feeding unit. PA-DED is the only WAAM process that can use powder along with the wire as feedstock (Table 4). It is characterized by higher deposition rates, greater energy concentration, better stability and less thermal distortion than in GTA-DED.

### 4.3. Binder Jetting (BJ)

The powder-based material is applied to the build platform with a recoater and then the print head selectively deposits the liquid adhesive on top, which both adheres the powder particles in the same layer and between the adjacent layers together. Following a layer, the product is lowered on the platform repeatedly to create more layers until the ‘green part’ with low strength and approximately 60% relative density is finished. Various materials can be utilized including polymers, ceramics and metals. The green part is then heated in a controlled atmosphere to remove the adhesive and to sinter the individual particles into a fully dense metal part.

The main drawbacks of binder jetting are the increase in post-processing time and it may not be the best choice for creating structural parts. The increase in density during sintering is obtained via shrinkage and loss of dimensional precision [86].

The primary advantage of binder jetting is that metal powder can be easily reinforced with ceramics as the process occurs at room temperature, which provides minimal distortion of parts associated with thermal effects.

### 4.4. Sheet Lamination (SL)

Sheet lamination with respect to metals is a process that binds layers using adhesive bonding, ultrasonic welding or friction stir welding [87,88] to form a single piece that is subsequently machined into the required part. However, sheet lamination has not found a broad application in the production of structural parts made of titanium alloys based on Ti_2_AlNb.

**Table 4:** Process parameters of MAM utilized for Ti_2_AlNb-based alloys.

MAM Category	Original AM Abbreviation	Feedstock Material	Feedstock Supply	Energy Source	Energy Source Parameters	Energy Spot Diameter, [mm]	Scanning Speed, [mm/s]	Layer Thickness [μm]	Atmosphere	Substrate
PBF	SLM [46,47,49,68]	Elemental powders	Recoater	Laser	p = 200–950 W, HD = 0.06–0.45 mm, VED = 60 J/mm^3^ *	0.08–0.7	300–1000	30–100	Argon	-
	SLM [11]	Pre-alloyed powder	Recoater	Laser	p = 80–280 W HD = 0.12 mm	-	600	30	Argon	Pre-heated at 200 °C
	SLM [64]	Pre-alloyed powder	Recoater	Laser	p = 80–280, HD = 0.12 mm VED = 30–170 J/mm^3^	0.08	200–1000	30	Argon	-
	SLM [13,70]	Pre-alloyed powder	Recoater	Laser	p = 140 W HD = 0.12 mm VED = 39–97 J/mm^3^	0.064	600	30	Argon	Pre-heated at 200 °C
	SLM [48,55]	Pre-alloyed powder + SiC whiskers	Recoater	Laser	p = 140 W HD = 0.12 mm VED = 34–78 J/mm^3^	0.12	850	30	Argon	Ti-6Al-4V substrate on Mo platform pre-heated at 200–980 °C
	LPBF [12,89]	Pre-alloyed and MAPS ** Powder	Recoater	Laser	p = 140 W HD = 0.12 mm VED = 34–78 J/mm^3^	0.12	850	30	Argon	Ti-6Al-4V substrate on Mo platform pre-heated at 200–980 °C
	SEBM [90]	Sputtered Al and Nb films	Direct current magnetron sputtering	Electron beam	I_b_ = 25 μA, V_a_ = 55 kV I_f_ = 466 μA ***	0.5	10	2	Vacuum	pure Ti
	SEBM [71]	Pre-alloyed powder	Recoater	Electron beam	I_b_ = 11–14.5 μA, LED = 36–48 J/mm ****	-	2800–4700	50	Vacuum	316L steel pre-heated at 850–900 °C
DED	LAM [10,52,53]	Pre-alloyed powder	Coaxial delivery nozzle	Laser	p = 1000 W	4	3	300	Argon	TA15
	LMD [51,59,60,61,62]	Pre-alloyed powder	Coaxial delivery nozzle	Laser	p = 1500–1700 W	3	3–4	300–350	Argon	Cold rolled Ti sheet/ *TC11/ TA15*
	LSF [50,57,58,91]	Pre-alloyed powder	Coaxial delivery nozzle	Laser	p = 1800–2000 W	3	4–6	350–400	Argon	Cold rolled Ti sheet /Ti60
	LMD [92]	Pre-alloyed powder	Coaxial delivery nozzle	Laser	p = 5000 W	6	13.3	900	Argon	Ti_2_AlNb plate pre-heated at 500 °C
	LMD [69]	Pre-alloyed powder	Coaxial delivery nozzle	Laser	p = 2000 W	4	8	800	Argon	TA15 plate
	LAW [20,65,66]	Pre-alloyed + TiB_2_ powders	Coaxial delivery nozzle	Laser	p = 1200–1500 W	3	5	-	Argon	Ti_2_AlNb plate
	TWPF [14]	Ø 2 mm TiNb wire Ø 1.6 mm Al wire	Two wire feeders Feeding angle—45°	Electron beam	I_b_ = 45 μA V_a_ = 60 kV I_f_ = 980 μA	-	4	2000	Vacuum	Ti-6Al-4V
	TEBF3 [93]	Ø 2 mm TiNb wire Ø 1.6 mm Al wire	Two wire feeders Feeding angle—45	Electron beam	I_b_ = 25 μA V_a_ = 60 kV I_f_ = 980 μA	-	3.7	-	Vacuum	Ti-6Al-4V
	DWAAM [67]	Ø 1.2 mm TiNb wire Ø 1.6 mm Al wire	Two wire feeders	Gas tungsten arc + Resistance heat power	U = 156 V I = 14.5 A Hot-wire current = 100 A	-	4	2000	Argon	Ti-6Al-4V
DED + point forging	PF-LD [54]	Pre-alloyed powder	Three direction co-axial powder delivery nozzle	Laser	p = 1584 W HD = 2.1 mm	3	6	500	Argon Flow	Ti-6Al-4V
BJ	BJ [45]	Elemental powders	Recoater	Reactive sintering	800, 1000, 1100 °C 6 h in vacuum furnace	-	-	100	Ambient	-

* *p*—laser power, HD—hatching distance, VED—volumetric energy density. ** MAPS—mechanically alloyed plasma spheroidized. *** *I_b_*—beam current, *V_a_*—accelerating voltage, *I_f_—*focus current. **** LED—linear energy density.

### 4.5. Hybrid Additive Manufacturing

In contrast, hybrid additive manufacturing (HAM) gains increasing interest in the production of metal parts with improved capabilities compared with standard DED and PBF processes. In [94], HAM is defined as a combination of two or more established manufacturing processes into a new, combined set-up. In [81], the HAM is divided into two groups.

The first group contains processes where two or more combined energy sources/tools result in a synergetic effect in the processing zone. The second group accounts for the processes, where the synergetic effects are obtained by a controlled combination of processes acting separately in order to fabricate parts in a more efficient and productive way, e.g., combination of DED and point forging (Table 3).

The features of production of Ti intermetallic alloys based on Ti_2_AlNb using AM are summarized in Table 4. First, relatively high energy is required for melting. Secondly, the low ductility of these materials can lead to the appearance of cracks caused by thermal stresses at high cooling rates [95]. Moreover, it leads to a low processability of the alloys at the ambient temperatures. Thirdly, Ti alloys are susceptible to contamination, when heated in oxygen atmosphere. Thus, the use of a protective atmosphere or vacuum is required for fusion.

DED and PBF are mainly used as they provide an optimal combination of mechanical properties with high manufacturability, i.e., capacity and minimal tolerance for machining. However, as can be seen from the Table 4, the process parameters vary in a wide range. Different supply schemes, rates and geometries of feedstock materials are utilized in MAM processes as discussed in the previous section. Finally, the substrate materials and the pre-heat conditions also vary widely, from pure Ti at room temperature to Mo heated at 1000 °C.

## 5. The Influence of MAM Process Parameters on Structure, Phase Composition and Mechanical Properties of the Alloys Based on Ti_2_AlNb

### 5.1. The Influence of MAM Process Parameters on Density and Microhardness

**Figure 6:**
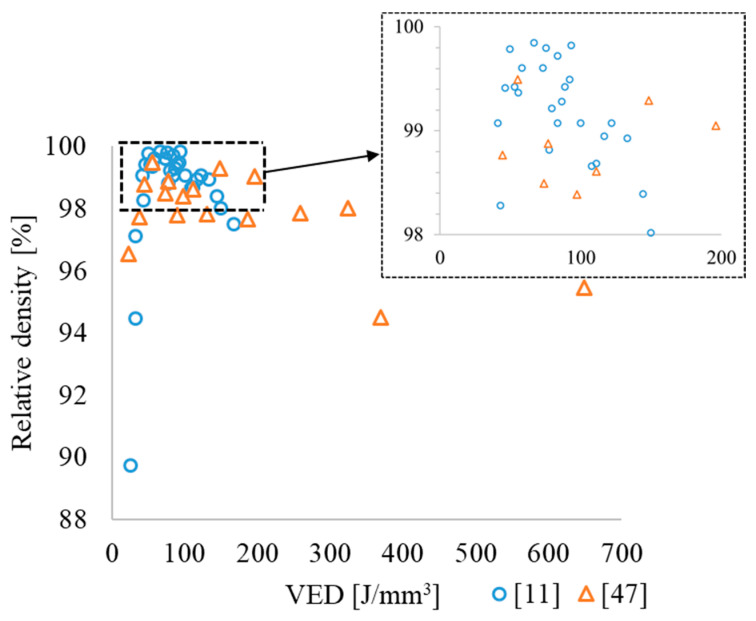
Influence of VED on the relative density of Ti_2_AlNb based alloy L-PBF coupons based on the data from [11,47].

Comprehensive research data showed the influence of AM processing parameters on the properties of as-built parts made of alloys based on orthorhombic titanium aluminides: density, hardness, elastic modulus, mechanical properties at room and elevated temperatures (650, 750 °C) and, in particular, the effect of volumetric energy density—VED =$\;\frac{P}{S\cdot HD\cdot L}$ [J/mm^3^], where laser power (P), scanning speed (S), hatch distance (HD) and layer thickness (L). Hatch distance and platform pre-heating temperature on density of the AM parts was evaluated for L-PBF (Figure 6).

According to previous research, the dependence of density on VED [11,47] (Figure 6), the maximum density (above 99.5%) is typical of alloys produced with VED of about 60 ± 10 J/mm^3^. This is explained in [47] by the fact that at a lower VED, the decrease in density and increase in porosity is associated with incomplete melting of the Nb powder due to insufficient energy input in L-PBF of a mixture of elemental Ti, Al and Nb powders. A similar effect was observed earlier in [96] during AlSi10Mg alloy manufacturing. A high VED resulted in overheating of the melt pool and evaporation of Al, which is in accordance with the previous studies of the L-PBF of TiAl [97]. When using the powder based on orthorhombic titanium aluminides [11], the density decrease with decrease in VED is associated with an increase in the viscosity of the melt pool, which hinders the escape of bubbles from the alloy in the liquid state. Increased VED leads to spattering and partial evaporation of the melt pool, which results in an increase in the fraction of pores [98,99]. Note that heating the platform to 200 °C [11,70] and 600–700 °C [12] provides the highest relative density of the fused material.

**Figure 7:**
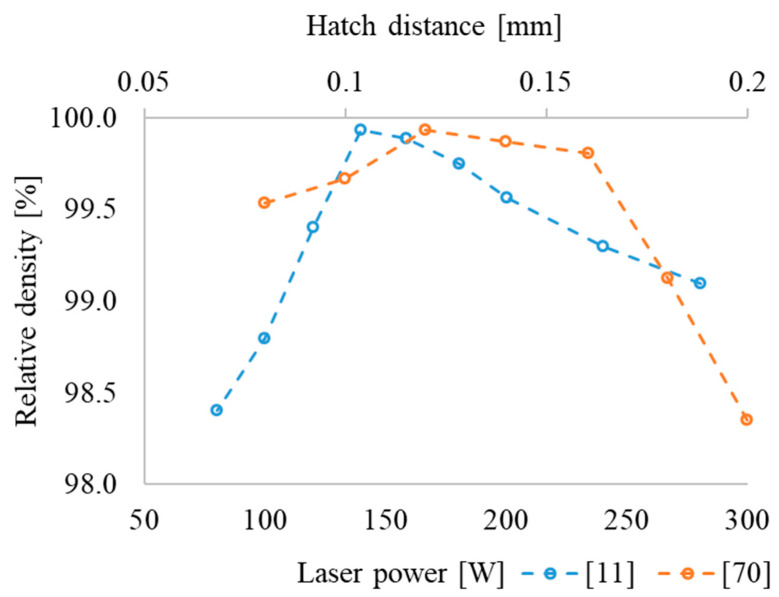
Effect of the laser energy (P) and hatch distance (HD) on the density of the O-phase-based alloy produced by L-PBF based on the data from [11,70].

Based on the analyzed data, it is still difficult to determine the optimal pre-heating temperature. However, the cracks can occur at pre-heating temperatures of 200 (MAPS), 200 and 500 °C (GA) in alloys manufactured from GA and MAPS powders. Moreover, the density was lower at pre-heating temperatures of 900 (MAPS), 980 °C (GA) [12]. Cracks in this case are associated with residual stresses that occur due to fast heating and cooling rates in MAM processes. The porosity vs. laser power dependence passes through a maximum at a certain energy and is similar to the effect of VED (Figure 7, dotted blue line).

In [70], the effect of hatch distance on density is considered. Figure 7 shows that the maximum relative density at a laser energy of 140 W as well as a minimal surface roughness that is typical of a hatch distance of 0.12 mm. A decrease or increase in HD leads to the formation of a “wavy” surface, which leads to porosity during fusion of the next layer. The optimal parameters of L-PBF, which provide fully dense samples, are summarized in Table 5.

The analysis of mechanical properties summarized in Table 6 allowed us to draw the following conclusions. The microhardness of the alloys based on Ti_2_AlNb of different composition vary in a wide range from 270 to 570 HV with possible spike up to 711 HV due to the addition of SiC whiskers during MAM [55]. The lower microhardness values of 270…300 HV are typical of the single-phase B2/β state, which is usually obtained by high cooling rates [10]. According to [100], the cooling rate during laser processing increases from 500 to 900 K/s as the laser power decreases from 2500 to 1000 W.

Increased microhardness of O-phase alloys is associated with the precipitation of second phases (O, α_2_, TiC) in the B2/β matrix. This occurs as a result of cyclic thermal [10,14,67,90] or thermomechanical [54] effects during MAM as well as during pre-heating of the platform at 500 °C and higher [12], subsequent heat treatment [46,47,56,60] and addition of SiC [55]. Note that the refinement of the precipitation of the second phases results in higher hardness [12,47].

The maximum microhardness of 570 ± 5 HV was obtained for a sample produced from Ti-24Al-25Nb-1Zr-1.4V-0.6Mo-0.3Si GA powder by L-PBF (at a platform temperature of 600 °C). This ensured 100% decomposition of the B2/β matrix with the formation of refined particles of the O-phase. In [92], the microhardness and elastic modulus of individual phases (O, B2, α_2_) were determined by nanoindentation and compared with the previous research [101,102,103]. The difference in the values of the microhardness in different studies can be associated with the different measurement conditions (load, holding time), in which different works vary within a wide range—from 0.3 N and 10 s [90] to 300 N and 30 s [54].

### 5.2. The Influence of MAM Process Parameters and Heat Treatment on Mechanical Properties at Room Temperature

Compressive mechanical properties of the of O-phase alloys were investigated in [14,55]. According to [14], the compressive properties of the Ti-22Al-25Nb alloy samples manufactured by TWPF method depend on the sample height due to differences in the phase-structural state (Table 6). The lower regions of the sample have lower strength with ductility compared to the upper regions of the sample. This can be explained by the formation of thinner and longer precipitates of O- and α_2_- phases in the upper regions compared to the bottom [14]. The compressive strength values of the composite obtained from Ti-24Al-25Nb-1Zr-1.4V-0.6Mo-0.3Si alloy powder with 0–15% SiC whiskers lie in the range of 2300 (0% SiC)—2550 MPa (5% SiC) [55]. Further increase in SiC above 5% resulted in the reduction of strength. According to [55], the increase in strength with the addition of SiC whiskers is associated with a refinement of the grain structure of the B2-matrix, due to the inhibition of grain growth by the precipitation of dispersed TiC particles. A further strength decrease is associated with the higher fraction of the B2/β matrix formed as a result of its stabilization by Si due to the dissolution of SiC-whiskers in the B2/β solid solution during melting.

Numerous results on tensile mechanical properties at room temperature have been obtained [10,11,12,14,20,51,52,54,56,59,62,64,65,66,67,70,93] and less research is devoted to elevated temperatures of 650 °C [13,20,62,64,66] and 750 °C [59]. A wide range of values characterizes the strength and ductility of MAM O-phase alloys—from ultimate tensile strength (UTS) and elongation (EL) of 220 MPa and 0% after L-PBF [12] to 1258 MPa after heat treatment [13] and 25.7% after DED accompanied by point forging [54].

The best combination of mechanical properties (UTS = 879 ± 107 MPa [14], EL = 19.3% [93]) of O-phase alloys manufactured by the WAAM method are lower than for L-PBF samples (UTS = 1090 ± 9.27 MPa, EL = 22 ± 0.48% [11]), UTS = 1144 MPa, EL = 24.25% [70], PF-LD (UTS = 1169 ± 10 MPa, EL = 25.7 ± 0.6% [54]. The main reason for the lower properties of WAAM alloys is the coarser-grained structure with the average B2/β-grain size exceeding 300 µm [14,67]. The average grain after L-PBF and DED-PF size did not exceed 40 μm [11,70] and 55 μm [54], respectively. Point forging provides the highest combination of properties with a relatively small dispersion [93], apparently due to the formation of a more uniform melt pool during manufacturing.

Previous research devoted to the mechanical properties of O-phase alloys (Table 6) manufactured using various types of powders (Table 6) showed that the use of a mixture of elemental metal powders does not provide a required combination of properties [49] due to embrittlement of the alloy as a result of oxygen contamination. An increased set of properties is provided by hot isostatic pressing (HIP) (1160 °C, 3 h) after MAM [104].

The best combination of tensile properties UTS = 1169 ± 10 MPa, EL = 25.7 ± 0.6% was obtained for Ti-21.18Al-25.3Nb-0.12O-0.1N alloy, manufactured by DED + PF. Thus, recrystallization after point forging results in the fine-grained B2/β structure [51]. A similar level of properties was obtained in [11,70] on the L-PBF Ti-22Al-25Nb alloy manufactured according to the parameters given in Table 4, which provides the maximum relative density and the formation of a fine-grained structure with an average grain size of 38 μm. At the same time, such a combination of properties is obtained in the considered alloys immediately after manufacturing in a metastable state with high content of the B2/β matrix (95% or more) that is thermodynamically unstable at elevated operating temperatures.

Annealing [11,49,66,104] or hardening heat treatment including quenching and aging [13,47,52,59,62,64,90] stabilizes the microstructure of the alloy due to the decomposition of the metastable B2/β-solid solution. However, the effect of hardening heat treatment on the mechanical properties of MAM alloys is uncertain (Table 6).

The effect of hardening heat treatment on the structure, phase composition and mechanical properties of the L-PBF Ti-22Al-25Nb alloy is considered in [13]. An increase in quenching temperature from 950 to 1050 and 1100 °C leads to a decrease in the YS from 949 to 900 MPa and EL from 24.9% to 12.1 and 14.3% with a maximum tensile strength of 981 MPa after quenching at 950 °C (Table 6). The observed ductility drop is associated with the precipitation of O, α_2_ -phases along the B2/β-grain boundaries, as well as the growth of the B2/β-grain after heating at 1100 °C (Table 6). At the same time, the volume fraction of the second phases after quenching is insignificant (no higher than 7%), but the precipitations are quite coarse. This does not allow obtaining higher strength in hardened alloys in comparison with the as-built almost single-phase B2/β state with a fine-grained structure with maximum solid solution strengthening. As shown in [13], during subsequent aging at 700 °C fine O-phase particles precipitate both in the body and along the B2/β-grain boundaries. This is accompanied by a decrease in EL down to 0.6–1.4% and contributes to the brittle fracture (after preliminary quenching from 1050, 1100 °C). At the same time, aging at 700 °C does not provide significant hardening, except for an alloy quenched from 950 °C (Table 6). An increase in the aging temperature up to 830 °C after quenching from 950 °C leads to the formation of coarser O, α_2_-precipitates, which provide a higher ductility of 6.1%. However, the strength properties correspond to that of as-built samples (Table 6). A similar tendency in mechanical properties of O-phase based alloys is also observed in other studies [64,93] (Table 6), which reveals that, at the moment, there are no heat treatment routes available that provide a strength-ductility combination higher than in an as-built state.

The authors of [70] considered the effects of the hatching distance (HD), inhomogeneity of the properties throughout the part height [10], the orientation of the sample on the building platform [52] and doping [20]. The superior properties for L-PBF were obtained at HD 0.12 and 0.16 mm in [70] (Table 6). The authors of [10] revealed an increase in strength and a decrease in ductility from the bottom to the top of the part (Table 6) due to a decrease in the volume fraction of embrittling O + α_2_-phases during L-PBF of Ti-22Al-25Nb alloy. A study of the mechanical properties of anisotropy of the MAM Ti-22Al-25Nb [52] after hardening heat treatment (quenching from 960 °C followed by aging 850 °C for 24 h) showed that the strength and ductility are slightly higher in the horizontal direction compared to the vertical one (Table 6). However, considering the dispersion of properties, the values overlap in both directions (Table 6). In [20], the effect of the TiB_2_ addition to Ti_2_AlNb powder on the properties of welded joints of L-DED Ti-22Al-25Nb alloy was investigated (Table 6). The highest strength and ductility of welded joints were achieved with a minimum fraction of TiB_2_ of 3.1%. An increase in the TiB_2_ content and coarsening of its particle size led to a drop in mechanical properties (Table 6) due to the negative effect of borides on the ductility of O-phase alloys.

### 5.3. The Influence of MAM Process Parameters and Heat Treatment on Mechanical Properties at Elevated Temperatures

Previous studies have shown that at a test temperature of 650 °C, the UTS and EL lie in the range of 365–833 MPa and 0–10%, respectively (Table 6). The lowest values of UTS (365 MPa and 375 ± 32.6 MPa) at an EL of 0.4% and 0.76 ± 0.05% were obtained in Ti-22Al-25Nb, Ti-24.8Al-22.3Nb alloys produced by L-PBF [13] and DWAAM [67] methods. In [13], the reason for the low mechanical properties may be due to residual stresses caused by the lack of platform pre-heating. The alloy in the as-built state consisted of a single-phase B2/β-solid solution in which decomposition took place with the formation of a dispersed O-phase, during heating to the testing temperature. A similar embrittlement effect (UTS = 400 MPa, EL = 0%) of an alloy with a B2 structure in an as-built state during tests at 650 °C was observed in [64]. On the one hand, the low combination of mechanical properties in [67] is probably associated with the large B2/β-grain size formed during the fusion of the alloy (the average size is about 350 μm, individual grains are up to 1.2 mm). On the other hand, it can be due to chemical inhomogeneity and Nb-rich zones, which stabilize the matrix B2/β phase. The highest combination of mechanical properties at 650 °C (UTS = 820–833 MPa, EL = 2.6–3%) was obtained at Ti–22Al–24Nb-0.5Mo [64] and a welded joint of the Ti-22Al-25Nb alloys [66] manufactured by L-PBF and L-DED, respectively. Similar results are characteristic of a structure consisting mainly of O-phase platelets and a minor fraction of α_2_ and B2 phases of the samples that were additionally heat treated (quenched from 1000 °C, aged at 800 °C, 24 h [64] and annealed at 850 °C, 2 h [66]).

High-temperature tests at 750 °C of Ti-22Al-25Nb alloy (B2 + α_2_ + O-phase structure) in [59] manufactured by LMD method and subjected to stress relief annealing at 550 °C, 2 h, followed by hardening heat treatment (960 °C, 3 h + 800 °C, 24 h) revealed a considerable spread of UTS in the range of 645–745 MPa, and EL of 1.5–3%. An increase in the test temperature by 100 °C from 650 to 750 °C had not resulted in a significant decrease in strength properties (Table 6). This is an encouraging result in terms of the use of MAM for O-phase alloys operating at temperatures in the range of 650–750 °C. Production of welded joints of O-phase alloys by the DED method using TiB_2_ additives [20] showed that the highest set of properties at a test temperature of 650 °C is obtained with a relatively small (3.1%) TiB_2_ addition. This provides a tensile strength of 638–640 MPa and elongation of 11.4–12.6% in the non-heat-treated state. At higher contents of TiB_2_, both strength and ductility decrease due to an increase in the embrittlement effect of TiB_2_, especially with the introduction of a coarse fraction (Table 6).

### 5.4. The Influence of MAM Process Parameters and Heat Treatment on Structure and Phase Composition

**Figure 8:**
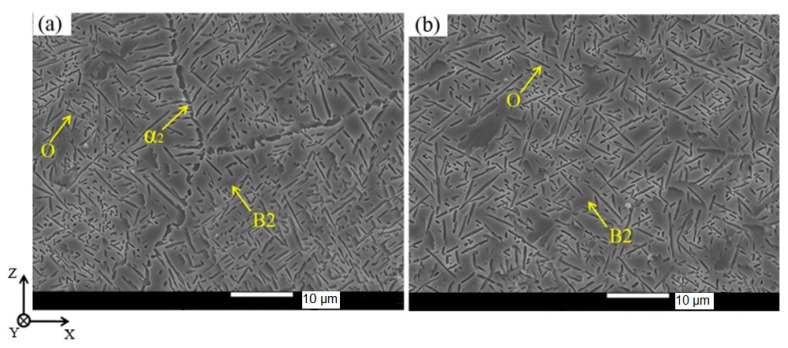
Typical microstructures of O-phase based Ti alloys produced by WAAM: (**a**) with grain boundary and (**b**) without grain boundary. Reproduced with permission from Elsevier [67].

The typical microstructure of O-phase alloys obtained by MAM consists of the B2/β-grains with a different fraction of grain boundary O- or α_2_-precipitates and intragranular precipitates of lamellar O-phase. Plate-like colonies grow mainly from the boundaries or appear in the form of individual small O-platelets between the colonies (Figure 8). The closer the pre-heating or processing temperature to the B2/β region, the lower the volume fraction of the second phases in the structure due to their dissolution and coagulation. Generally, the O and α_2_ precipitates at the grain boundary dissolve in the last turn. The B2/β-grains grow (Table 6) when the fraction of the O and α_2_—phases decrease upon transition to a single-phase B2/β—region during post-processing. Chemical inhomogeneity of alloying elements in as-built state can take place if a mixture of elemental powders as well as Al and Ti-Nb alloy wires are used as feedstock materials. Thus, regions enriched with refractory elements primarily Nb on the one hand and with a more fusible Al on the other hand are formed.

The phase composition of the parts or regions of inhomogeneous elementals distribution in as-built state and after post-processing is determined by four main factors: (1) chemical composition of the analyzed alloy (region); (2) heating temperature; (3) holding time; and (4) cooling rate. In the 1st factor, the chemical composition allows for choosing the binary (Ti-Al, Ti-Nb) or ternary Ti-Al-Nb (Figure 2 and Figure 3) diagram to evaluate possible changes in the phase composition depending on the temperature of the final heat treatment (the 2nd factor). For more complex alloy compositions of Al, the Nb equivalents mentioned in Section 1 can be employed. The 3rd and 4th factors allow for the evaluation of the completeness of phase transformations at the heating temperature in accordance with the phase diagram. Lower heating temperatures and holding times with higher cooling rate result in incomplete phase transformation.

**Figure 9:**
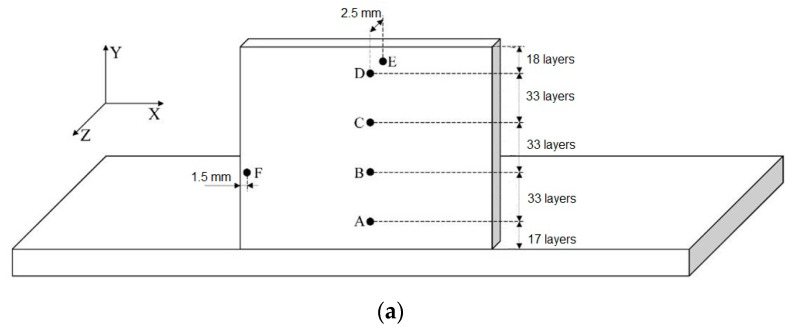

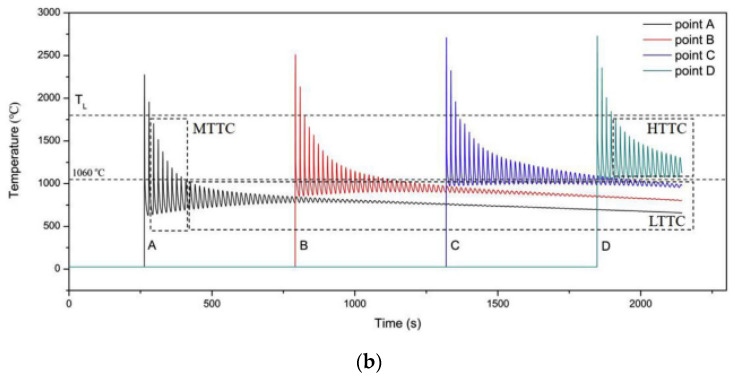
Thermal history in different points through the height of the sample produced by L-DED (**a**) used to simulate the time-temperature (**b**). Reproduced with permission from Elsevier [10].

Generally, the solidification texture <001> _B2/β_ typical of BCC metals is formed along building direction of MAM products [105]. The platform/substrate, powder pre-heating temperature, hatch distance and other printing parameters affect the crystallization texture. The simulation of time–temperature dependence during L-DED at different points throughout the sample height (Figure 9) was conducted in [10]. The temperature change at each point of the synthesized O-phase alloy is characterized by a self-extinguishing oscillating amplitude of temperature. In a high temperature thermal cycle (HTTC), the heating temperature exceeds β_tr_ (1060 °C). For the low temperature thermal cycle (LTTC) the heating temperature is below β_tr_. When β_tr_ belongs to the temperature range between the highest and the lowest, the medium temperature thermal cycle (MTTC) takes place. The experiment in [10] proved that the thermal “history” in each region of the fused sample determine the final phase composition of this region.

## 6. Perspectives of MAM for Production of Critical Parts Made of Ti_2_AlNb Based Alloys

Manufacturing O-phase-based alloys via MAM is still at the initial stage of its development; therefore, many technologic aspects still need to be clarified. To date, there are no studies, except [71], on the use of the EB-PBF method to produce O-phase alloys. The main reason for that is the lower availability of machinery compared with L-PBF due to the limitations associated with the original patent by Arcam, which expired in 2015. In addition, only Ti-48Al-2Cr-2Nb γ-aluminide powders by Arcam are commercially available. Therefore, the adaptation of process parameters for a new powder composition is associated with significant costs [106]. However, EB-PBF can be promising research direction because EB-PBF proposes a number of the advantages compared with L-PBF of O-phase-based alloys: (1) low contamination by interstitials associated with high vacuum in build chamber; (2) absence of residual stresses of the as-built parts due advanced principle of material pre-heating; and (3) higher deposition rates.

According to [14,67], the twin wire feeding methods provide the most promising results among the WAAM processes for O-phase alloys. However, the resulting properties of the alloys are lower than of the alloys produced with feedstock powder processes L-PBF and a hybrid additive manufacturing method combining L-DED and point forging [11,54,70]. The development of WAAM technologies to produce O-phase alloys, in our opinion, is primarily associated with obtaining a fine-grained structure and a homogeneous composition of the alloy.

Powder technologies, such as L-PBF and combination of L-DED + point forging, make it possible to produce alloys with excellent mechanical properties at room temperature (Table 6) in a thermodynamically non-equilibrium state. However, it is necessary to continue research on the development of heat treatment and thermomechanical processing that provide a combination of a stable structural-phase state with required operating properties at elevated temperatures.

DED processes (DMD, LSF, LDM) were used to obtain bimetallic composites with pure Ti, Ti alloys, O-phase alloys and gamma aluminides [50,51,59,60,61,62,69,91,92]. DED (LAW) technology was used to obtain welded joints of the O-phase [20,65,66]. The issues of high-quality feedstock materials and post-processing selection to provide a sufficient margin of strength and ductility at room and elevated temperatures have not been solved (Table 6).

There are no studies on the relationship between the structure and texture of O-phase alloys for the considered MAM processes. The performance of the alloys produced by MAM needs to be estimated, namely low- and high-cyclic fatigue, impact toughness, crack resistance, fracture toughness at room temperature and at elevated operating temperatures of 600–700 ° C (creep and heat resistance, etc.).

There is also a questionable issue on the chemical composition of the O-phase alloys, both in terms of the optimal ratio of the main alloying elements (Al, Nb) and additional alloying elements and impurities.

**Figure 10:**
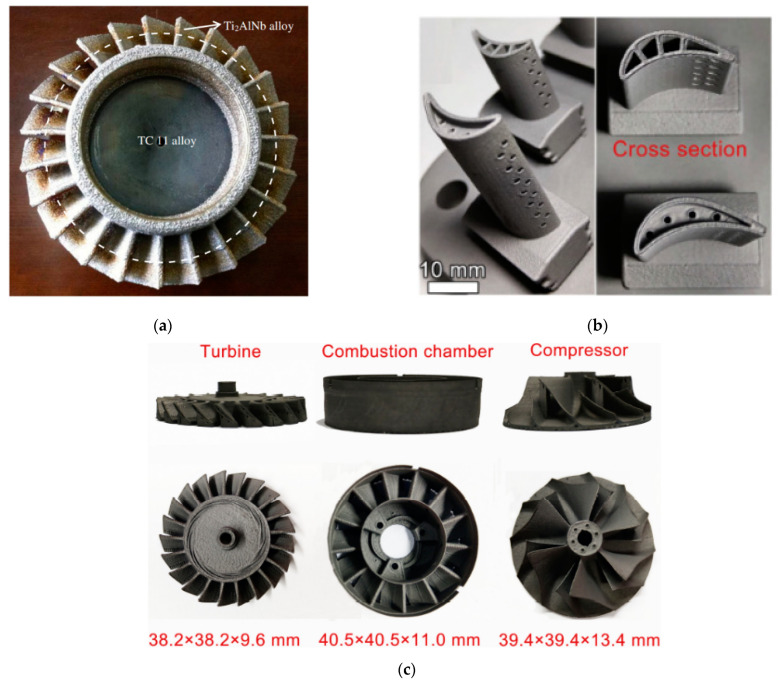
Prototypes of MAM parts of O-phase alloys. Reproduced with permission from Elsevier [62] (**a**), [11] (**b**), [70] (**c**).

Future work on obtaining the reliable and stable properties will enable to produce high-quality O-phase alloys parts by MAM and to replace the other refractory Ni-based alloys and Ti alloys based on gamma-aluminide. Prototypes of such products have already been presented in [11,62,70] (Figure 10).

## 7. Conclusions

This review investigates issues related to the general characteristics of currently used intermetallic alloys based on orthorhombic titanium aluminide: alloying elements, impurities, phase composition, structure, mechanical properties and phase transformations in various temperature ranges along with the feedstock materials (wire and powder), process classification and machines used for metal additive manufacturing of O-phase alloys. Based on this comprehensive data analysis we conclude:

- L-PBF, DED and WAAM are the most widely used metal additive manufacturing processes to produce O-phase alloys. MAM provides fully dense, fine-grained material with a superior combination of mechanical properties at room temperature. However, a thermodynamic equilibrium is not reached in the as-built state.
- Post-processing that provides a thermally stable structure with balanced properties at room and elevated temperatures has not yet been developed.
- Among the WAAM processes for O-phase alloys, the twin feeding methods have the greatest potential interest. For powder feedstock, the L-PBF and hybrid additive manufacturing processes combining the L-DED and point forging demonstrated excellent mechanical properties.
- Due to the increased porosity and post-processing time, the binder jetting process may not be the best option for creating structural parts of O-phase alloys.
- Further research can be focused on a detailed study of the influence of post-processing and chemical composition on the formation of the microstructure and mechanical properties including cyclic loading, fracture toughness and creep tests. This ensures the development of heat treatments, which will provide a combination of properties, that can compete with refractory nickel, titanium and titanium gamma aluminides at operating temperatures in the range of 600–700 °C.

## Figures and Tables

**Table 3:** Classification of the metal additive manufacturing (MAM) processes based on AM category according to ISO/ASTM 52900–2021, feedstock material and energy source and examples of terms employed.

MAM Category	Feedstock Form	Energy Source	Variety of Terms
Powder Bed Fusion	Powder	Laser	Selective Laser Sintering (SLS) Selective Laser Melting (SLM) Direct Metal Laser Sintering (DMLS) Laser beam melting (LBM)
	Powder	Electron Beam	Selective electron beam melting (SEBM) Electron Beam Melting (EBM) Electron-beam additive manufacturing (EBAM)
Direct Energy Deposition (Direct Metal Deposition) 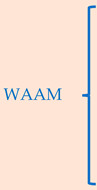	Powder Wire	Laser	Laser additive manufacturing (LAM) Laser solid forming (LSF) Direct metal tooling (DMT) Laser engineered net shaping (LENS) Laser-additive welding (LAW) Laser deposition manufacturing (LDM) Laser metal deposition (LMD) Laser metal direct forming(LMDF)
	Wire	Electron Beam	Electronic Beam Freeform Fabrication (EBF3) Electron-beam additive manufacturing (EBAM) Twin-wire electron beam freeform fabrication (TEBF3)
	Powder	Plasma arc	3D plasma-metal deposition (3DPMD) Plasma deposition manufacturing (PDM)
	Wire		Twin-wire arc additive manufacturing (TWAAM) Twin-wire welding-based additive manufacturing (TWAM) Double-wire arc additive manufacturing system (DWAAM)
	Wire	Gas tungsten arc	Gas tungsten arc (GTA) Gas tungsten arc welding (GTAW) Tungsten Inert Gas (TIG)
	Wire	Gas metal arc	Gas-Shielded Metal Arc Welding Gas metal arc welding-based additive manufacturing (GMAW-AM)
Binder Jetting	Powder	Furnace heating	Binder jetting (BJ)
Sheet Lamination	Sheets	Sonotrode, Friction stir welding	Laminated Object Manufacturing (LOM) Computer-Aided Manufacturing of Laminated Engineering Materials (CAM-LEM) Composite Based Additive Manufacturing (CBAM) Ultrasonic Additive Manufacturing (UAM)

**Table 5:** L-PBF parameters providing minimal porosity of orthorhombic titanium aluminides.

Alloy Composition [at. %]	P [W]	V [mm/s]	HD [µm]	h [µm]	VED [J/mm^3^]	Density [%]	Pre-Heating [ °C]	Ref.
Ti-22Al-25Nb	200	1000	60	60	55.6	99.55	-	[47]
Ti-22Al-25Nb	140	600	120	30	64.8	99.93	200	[11,70]
Ti-22Al-24Nb-0.5Mo	180	1000	100	30	60	99.8	-	[64]
Ti-16Al-22Nb-0.1Mo-0.3Hf-0.3Ta-1.5Zr-0.8Si-0.9Fe	140	650	120	30	59.8	99.6	900	[12]
Ti-24Al-25Nb-1Zr-1.4V-0.6Mo-0.3Si	140	850	100	30	54.9	99.92	700	[12]

**Table 6:** Physical and mechanical properties of AM alloys based on orthorhombic titanium aluminides produced by MAM.

MAM Category	Original AM Abbreviation	Alloy	Processing Conditions/Sampling					Phase Composition	HV [kgf/mm^2^]/E [GPa]	UTS/YS * [MPa]	EL ** [%]	UTS [MPa] at 650 °C	EL [%] at 650 °C	Grain Size [μm]/Comments	Refs.
PBF	SLM	Ti-22Al-25Nb	as-built					90%B2 + 10%(α_2_ + O)	338 ± 7.4		-	-	-	-	[46]
			as-built + annealing 1350 °C					80%B2 + 20%O	358 ± 5.8	286					[49,68]
			as-built + annealing 1350 °C + aging 700–1100 °C					B2 + O + α_2_	360–420						[47]
	L-PBF	Ti-24Al-25Nb-1Zr-1.4V-0.6Mo-0.3Si (GA powder)	pre-heating 200 °C					B2	392	-	-	-	-	-	[12,48,56]
			pre-heating 500 °C					B2 + O_weak_	512	-	-				
			pre-heating 600 °C					O	568	220	-				
			pre-heating 700 °C	As-built				O	525–568	300–450	-				
				ST 950 °C				B2 + O	395	690	-				
				ST 1050 °C				B2 + O	384	-	-				
				HIP 1160 °C				B2 + O	360	1030	1.2				
			pre-heating 980 °C					22%O + 78%B2	453	693	-				
		Ti-16Al-22Nb-0.1 Mo-0.3Hf-0.3Ta-1.5Zr-0.8Si-0.9Fe (MAPS powder)	pre-heating 200 °C					B2	405	-	-				
			pre-heating 700 °C					48%O + 52%B2	435	-	-				
			pre-heating 900 °C					28%O + 72%B2	426	-	-				
		Ti-24Al-25Nb-1Zr-1.4V-0.6Mo-0.3Si (GA powder) + SiC	0% SiC					O	395 ± 10	2300 ± 120	-	-	-	-	[55]
			5% SiC					O + B2 + TiC	577 ± 17	2550 ± 170	-				
			10% SiC					O + B2 + TiC	701 ± 20	2420 ± 100	-				
			15% SiC					O + B2 + TiC	711 ± 15	2370 ± 150	-				
	SLM	Ti-22Al-25Nb	As-built					B2 + O	-	1090 ± 9 / 960 ± 11	22.7 ± 0.5	-	-	38	[11]
	SLM	Ti–22Al–24Nb-0.5Mo	As-built					B2	-	~890	10	400	-	-	[64]
			ST 880 °C		aging 800 °C 24 h			O + α_2_		~720	2.5	-	-		
			ST 920 °C							~685	6	690	6		
			ST 960 °C							780	2.8	-	-		
			ST 1000 °C							~810	3.4	820	3		
			ST 1090 °C							~735	2.3	-	-		
	SLM	Ti-22Al-25Nb (Ti–18.58Al–25.59Nb)	As-built					99.8%B2 + 0.2%O	-/101	973/949	24.90	365	0.4	42.8	[13]
			ST 950°C					93%B2 + 5%O + 2%α_2_	-/101	981/932	12.1	560	2.1	42.2	
			ST 1050°C					94%B2 + 5%O + 1%α_2_	-/105	952/930	14.3			45.7	
			ST 1100°C					98%B2 + 2%O	-/100	943/900	12.3			284	
			ST 950°C + 700 °C					51%B2 + 46%O + 3%α_2_	-/113	1258/1250	1.4	749	1.6	17.7	
			ST 1050°C + 700 °C					82%B2 + 17.5%O + 0.5%α_2_	-/122	-/1027	0.9			44.7	
			ST 1100°C + 700 °C					93%B2 + 6%O + 1%α_2_	- /118	-/653	0.6			243.8	
			ST 950°C + 830 °C					80%B2 + 12%O + 8%α_2_	-/112	978/866	6.1	611	10	40.1	
	SLM	Ti–18.58Al–25.59Nb	As-built		HD = 0.08			B2/β	-	~1045/885	~16	-	-	69.3	[70]
					HD = 0.12					~1075/960	~23			45.5	
					HD = 0.16					1144/981	24.25			35.7	
					HD = 0.2					~950/881	~9.5			31.8	
	SEBM	Ti-19.4Al-13.5Nb	-					O + β/B2	570	-	-	-	-	-	[90]
		Ti-22Al-25Nb	As-built					O + β/B2 + α_2_	295–345	1060 ± 24/890 ± 43	3.67 ± 1.15	-	-	104 ± 33	[71]
			HIP 1030 °C, 150 MPa, 3 h						~390	1101 ± 23/934 ± 43	3.5			106 ± 33	
DED	LAM	Ti-22Al-25Nb	As-built Through height		30–40 mm			B2	300–270	1200	6	-	-	-	[10]
					20–30 mm			B2 + O + α_2_	320–280	1136	4.5				
					10–20 mm			O + α_2_ + B2	350–385	892	4				
					0–10 mm				350–370	721	5				
			ST 960 °C +850 °C, 24 h		Vertical				-	981 ± 21	4.5 ± 1.8	-	-	-	[52]
					Horizontal					1017 ± 16	5.8 ± 0.7				
	LMD	Ti-22Al-25Nb	As-built		-			B2+ O + α_2_	-	941 ± 5	1.5 ± 0.3	-	-	-	[51,59,60,62]
					940 °C, 2 h				352	-	-	-	-		
					940 °C, 0.5 h + 760 °C. 12 h				429	-	-	-	-		
			As-built + 550 °C. 2 h		-				-	976; 1100; 1107	2; 2; 2.5	-	-		
					960 °C. 1 h + 560 °C, 24 h				-	1051; 1003; -	1; 1; -	580	-		
					960 °C. 2 h + 750 °C, 3 h				-	1103; 1041; 891	0.5;-;-		-		
					960 °C. 3 h + 800 °C, 24 h				-	979; 998; 1060	1; 2.5; 2	645; 715; 745 (at 750 °C)	1.5; 3; 2		
	LSF	Substrate Ti + Ti_2_AlNb	Weld joint					O → α + β →α + α′ → α′→ α + β → α + β/B2 + α_2_ → β/B2 + α_2_ →β/B2 → B2 + α+O →B2	170(α)–470(O)	-	-	-	-		[50,91]
		Ti-20Al-27Nb	As-built					Top: B2 Bottom: B2 + O	375–525					20–400	
		Ti-22Al-27Nb						B2 + O between β-dendrites	460–830					5–80	
	LDM	Bimetal TA15 + Ti_2_AlNb	As-built					-	-	893	5.5	-	-	Fracture in transition zone from Ti_2_AlNb	[69]
			As-built + ST 900 °C. 1 h							909	6.7				
			As-built + ST 900 °C. 1 h+ + 800°C. 4 h							833	6.1				
	LAW	Ti-22Al-25Nb welded by Ti-21Al-23Nb-1Mo	HAZ					α_2_ + B2	-	1282	5.6	980	7.9	-	[20,65,66]
			Weld joint					B2		1032	7.2	820	6.0		
			Annealing 850 °C. 2 h					O + B2		1066	-	833	2.6		
			Annealing 1000 °C. 2 h					O + B2		926	-	740	5.8		
			+ TiB_2_ powder	Fine			3.1%	-		989.3	5.7	638.1	12.6		
							6%			966.5	3.9	623.1	7.9		
							16.1%			944.1	4.0	581.3	5.1		
							24.3%			918.0	4.3	557.5	4.2		
				Coarse			3.1%			942.7	4.9	640.6	11.4		
							6%			771.8	-	614.4	3.3		
							16.1%			590.3	-	613.2	2.4		
							24.3%			614.7	-	638.1	2.0		
	DWAAM	Ti-24.8Al-22.3Nb	-					β/B2 + α_2_ + O	335 ± 28	504 ± 38.59	0.41 ± 0.03	375 ± 32.6	0.76	800–1200	[67]
	TWPF (DWAAM)	Ti-22Al-25Nb	top					38%β/B2 + 7%α_2_ + 55%O	454 ± 10	(c): 2123 ± 16.5 (p): 880 ± 107.5	(c): 27.7 ± 1.49 (p): 1.05 ± 0.23	-	-	350/ c–center p–periphery	[14]
			bottom					84.5%O + 15.5%β/B2	414 ± 12	(C):1972 ± 89 (p): 600 ± 8.4	(C):26.5 ± 0.34 (p): 0.45 ± 0.02				
	TWAAM TEBF3	Ti-22Al-25Nb	SSF					-	-	507; 537; 662	2.4; 4.2; 11.1	-	-	-	[93]
			DSF							600; 300; 650	19.3; 1.7; 7.5				
			PF ***							699; 764; 659	8.2; 8.3; 8.6				
	PF-LD	Ti-21.21Al-25.35Nb-0.11O-0.08N	LD					O + β/B2 + α_2_	295–310	927 ± 7.5/845± 6.6	6.9 ± 0.9	-	-	350	[54]
		Ti-21.18Al-25.37Nb-0.12O-0.1N	PF-LD			t–top		O + B2	322–354	1050 ± 9.5/1041 ± 8	20.8 ± 0.4			71–120	
						b–bottom				1169 ± 10/1041 ± 8	25.7 ± 0.6			55–49	

* UTS—ultimate tensile strength; YS—yield strength; ** EL—elongation at failure; *** double-side-feeding (DSF); single-side-feeding (SSF); parallel-feeding (PF).

## Data Availability

Not applicable.
